# High-brightness laser imaging with tunable speckle reduction enabled by electroactive micro-optic diffusers

**DOI:** 10.1038/s41598-017-15553-9

**Published:** 2017-11-10

**Authors:** Hamid Farrokhi, Thazhe Madam Rohith, Jeeranan Boonruangkan, Seunghwoi Han, Hyunwoong Kim, Seung-Woo Kim, Young-Jin Kim

**Affiliations:** 1 School of Mechanical and Aerospace Engineering, Nanyang Technological University, 50 Nanyang Avenue, Singapore, 639798 Singapore; 2 Wellman Center for Photomedicine, Massachusetts General Hospital and Harvard Medical School, Boston, Massachusetts 02114 USA; 3 Department of Mechanical Engineering, Korea Advanced Institute of Science and Technology (KAIST), Daejeon, 34141 Republic of Korea

## Abstract

High coherence of lasers is desirable in high-speed, high-resolution, and wide-field imaging. However, it also causes unavoidable background speckle noise thus degrades the image quality in traditional microscopy and more significantly in interferometric quantitative phase imaging (QPI). QPI utilizes optical interference for high-precision measurement of the optical properties where the speckle can severely distort the information. To overcome this, we demonstrated a light source system having a wide tunability in the spatial coherence over 43% by controlling the illumination angle, scatterer’s size, and the rotational speed of an electroactive-polymer rotational micro-optic diffuser. Spatially random phase modulation was implemented for the lower speckle imaging with over a 50% speckle reduction without a significant degradation in the temporal coherence. Our coherence control technique will provide a unique solution for a low-speckle, full-field, and coherent imaging in optically scattering media in the fields of healthcare sciences, material sciences and high-precision engineering.

## Introduction

High-speed and high-resolution optical imaging through scattering optical media, e.g. biological tissues, plasmonic nanoparticles, and non-ideal optics with surface roughness or contamination, has been highly requested in the field of biological science, material science, medical diagnosis, and precision engineering. These commonly require a high brightness light illumination over a wide field-of-view, which can get benefits by introducing coherent lasers as the light source. However, the random scattering in imaging systems caused by coherenet laser field works as the primary obstacle for the optical imaging. It is because the coherent artifacts, such as speckle, diffraction, and intensity overshoot around the edges, degrade the image quality severely in both tradiational non-coherent imaging and coherenent quantitative phase imaging (QPI). In QPI, optical interference, enabled by the high degree of coherence of the lasers, superimposes unwanted background speckles so significantly hinders the successful phase reconstruction^1–6^. Thus, extensive research works on coherence control of light sources have been addressed^1–24^. The coherence can be split into two, the temporal and spatial ones. The temporal coherence, which is the prerequisite for the optical interferometry, causes unwanted scattered or diffracted beams from dusts, particles, roughness, or defects on the surface of optical components and makes them to interfere with the measurement beam; this usually generates stationary low-frequency noises in the spatial domain. On the other hand, the spatial coherence, required for the interferometric imaging over a large field-of-view (FOV), causes high-frequency speckle noises, which degrade overall image quality and measurement precision^7,8^. For the best biomedical laser imaging without speckle, diffraction, and edge effects, there have been considerable research works conducted to suppress the speckle noise in interferometric imaging^1–24^.

**Figure 1:**
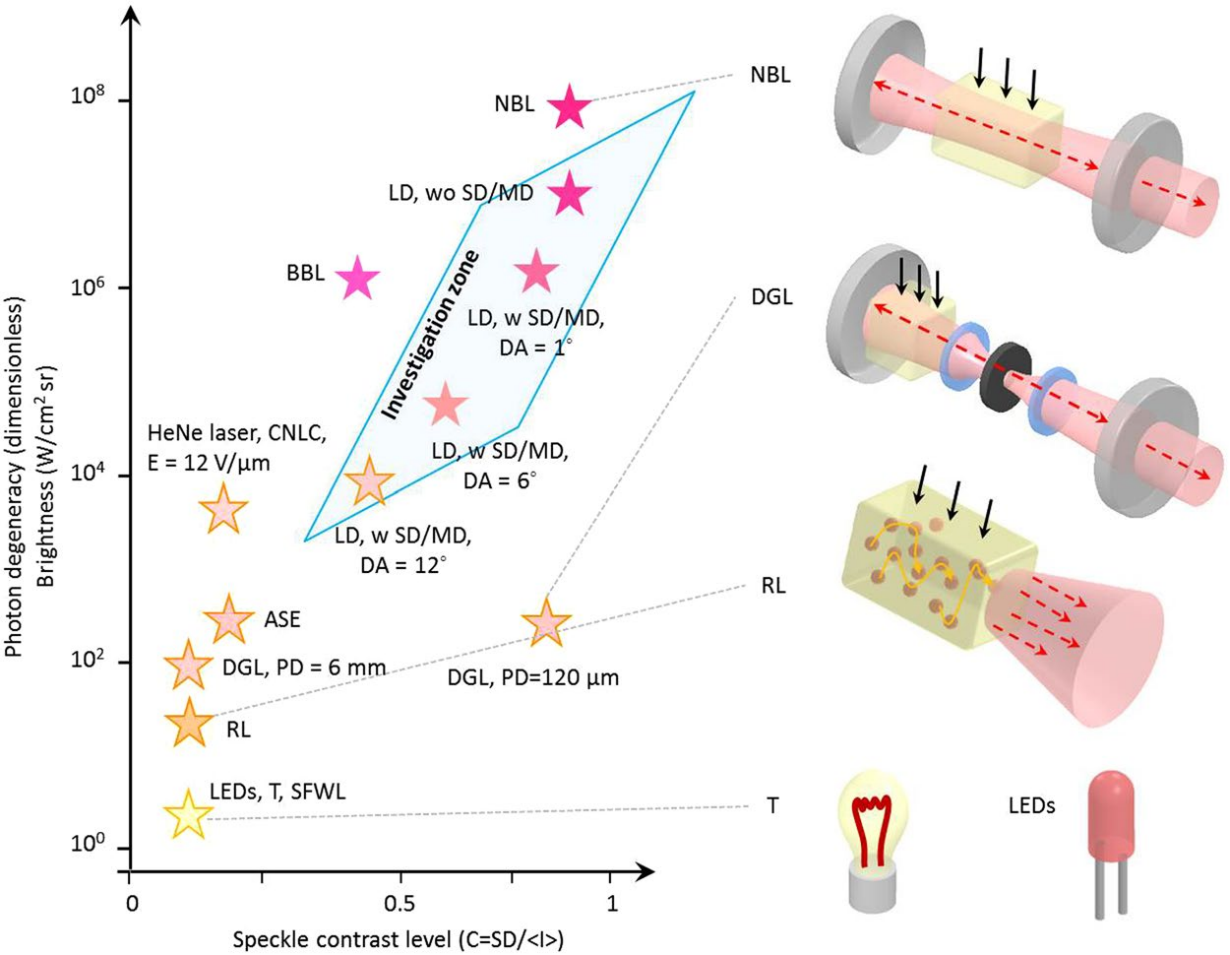
State-of-the-art light sources for optical imaging; classified by photon degeneracy and speckle contrast level. ‘Investigation zone’ indicates the tunable temporal and spatial coherence area demonstrated in this investigation. NBL: narrowband laser^21,30^, BBL: broadband laser^21,29,31^, ASE: amplified spontaneous emission^21,22^, RL: random laser^21,22^, LEDs: light emitting diodes^21,30,32^, T: thermal sources^21,30,32^, SFWL: spatially filtered white light^21,28,30,32^, DGL: degenerate laser with pinhole inside the cavity^23^, CNLC: chiral nematic liquid crystal^19^, LD w/wo SD/MD: a laser diode with and without a static diffuser (SD) and a moving diffuser (MD) with a specific diffusion angle (DA).

Thermal light sources (halogen, xenon, and mercury lamps) and light emitting diodes (LEDs) have been widely used as the partially coherent light sources after the spatial filtering through a small-diameter pinhole (∼5–50 μm); spatial filtering provides a higher spatial coherence so enables the formation of higher visibility interferograms over a larger FOV. However, this improvement in the spatial coherence is accompanied by severe optical power attenuation (from several W to a few mW). Therefore, a bright coherent laser illumination with suppressed coherent artifacts is highly requested for efficient optical imaging of light-sample interaction in optically turbid media having micro/nano scatterers inside. Previous studies for speckle noise control are summarized in Fig. 1, with the speckle contrast level (related to spatial coherence) as the horizontal axis and the photon degeneracy (related to temporal coherence and brightness) as the vertical axis. High photon degeneracy (δ), the number of photons per a unit coherence volume, is crucial for an efficient light-sample interaction hence fast and precise measurement. Coherent photons from different volumes cannot interfere with one another so do not generate speckles; therefore, a light source with many bright coherence volumes enables speckle-free sensitive imaging over a large FOV. In this point of view, the limitations of low-coherence light sources and coherent lasers are clear. Low-coherence light sources suffer from the small number of photons per coherence volume (low photon degeneracy) as shown as T, SFWL, and LEDs in Fig. 1. On the other hand, conventional lasers having a large number of photons per unit volume (high photon degeneracy) generate unexpected coherent artifacts as shown as NBL in Fig. 1. These are the two extreme cases located in the left bottom (T, SFWL, and LEDs) and right top (NBL) of the Fig. 1. As the alternatives to the low-coherence light sources, broadband laser-like sources, such as amplified spontaneous emission (ASE) sources and super luminescent laser diodes (SLDs) have been adopted^20^ as shown as ASE in Fig. 1; however, they still preserves a high-level spatial coherence so coherent speckles cannot be sufficiently reduced^21^. In order to suppress the effective spatial coherence of the conventional lasers, rotating optical diffusers^16^, colloidal solutions^17^, micro electro-mechanical mirrors^18^, or chiral nematic liquid crystal diffusers^19^ have been introduced to the illumination path; these techniques, however, requires mechanical moving parts generating vibration noise or a longer acquisition time, which mitigates the advantages (See CNLC in Fig. 1). The concepts of random lasers^21,22^, and degenerate laser^23^, were recently demonstrated as the promising means to generate an intermediate degree of spatial coherence as shown as RL and DGL-PD (6 mm and 120 μm) in Fig. 1; however, they can be realized with a high lasing threshold, a low collection efficiency, a large footprint, and a high cost. Because the scattering environment differs in each biomedical imaging application, the ideal light source for the best imaging should have active tunability in the coherences, which has remained as a difficult task to be resolved. At the same time, it should be realized in a simple, cost effective, high-speed, and vibration-free manner.

In this investigation, we report a simple, stable, vibration-free, cost-effective, and tunable approach to control the laser coherence for efficient speckle reduction in quantitative phase imaging. This was realized by actively tailoring the spatial coherence of coherent continuous-wave (CW) lasers via a combination of static and moving electroactive polymers having a large number of micro-optical scatterers (10, 30 and 100 μm) on it, which enables high-speed wavefront control of the incidence laser beam. The proposed method reduced the background speckle noise by 50% over the full FOV, while decreasing the speckle contrast in coherent imaging under a highly scattering media from 0.99 to 0.49. This will enable a high brightness, high speed, high sensitivity, and quantitative bio-medical and material imaging over a large FOV.

## Results and Discussion

### Speckle Noise Reduction in Laser Imaging by Electroactive Micro-Optic Diffusers

**Figure 2:**
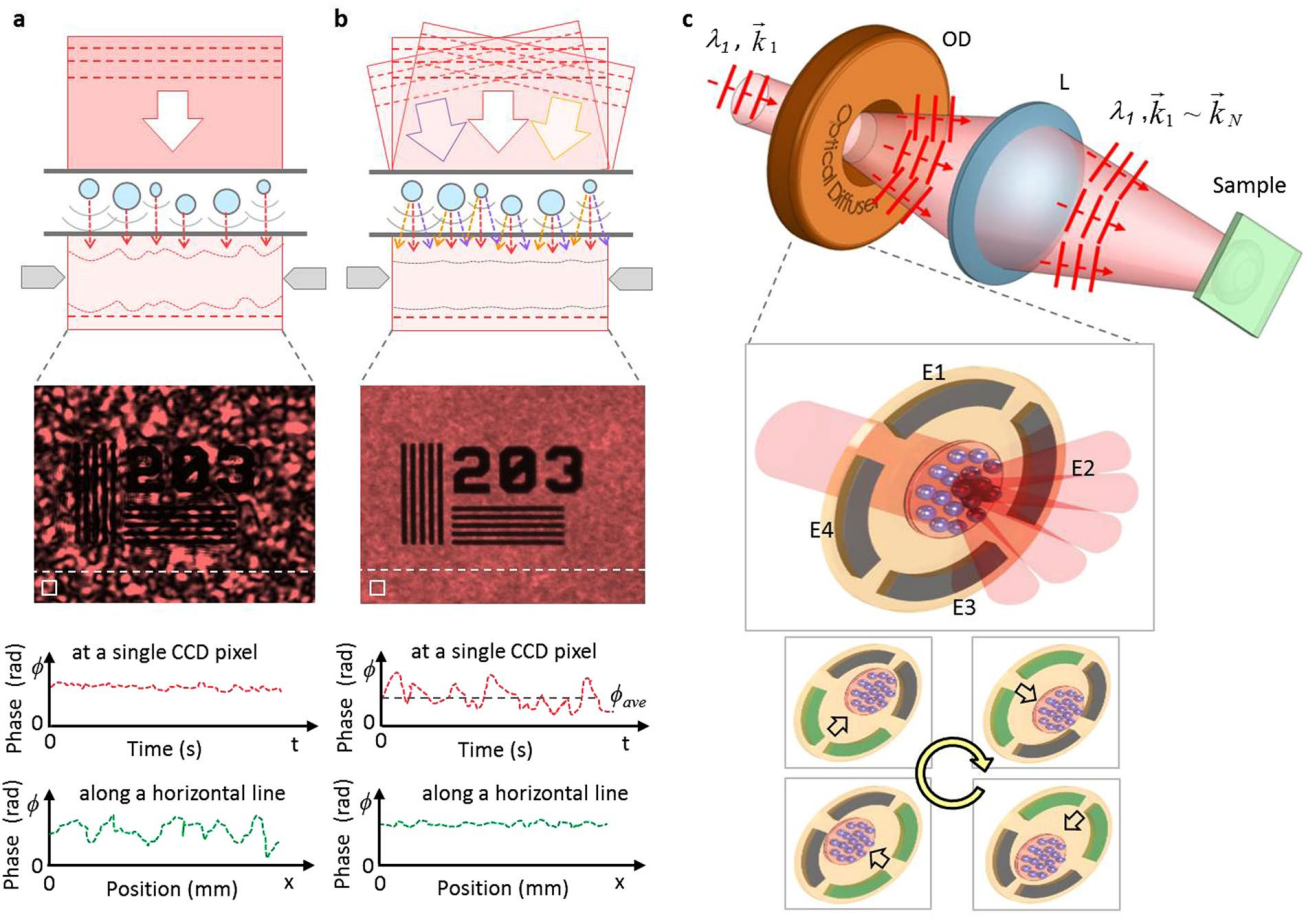
Electroactive optical diffuser for low-speckle laser imaging. (**a**) Spatially coherent illumination with well-defined wavefronts causes strong background speckle. (See a position-dependent spatial phase variation along a horizontal white dash line). (**b**) Temporal wavefront control over time averages out the coherent speckles in the spatial domain. (See a time-dependent phase variation at a CCD pixel). (**c**) Conceptual image of speckle reduction using an electroactive optical diffuser, with four electrodes (E1, E2, E3, and E4) for rotational diffusion angle control. OD: optical diffuser and L: lens.

High degree of coherence, on the other hand, accompanies unexpected background speckles across overall image so causes a severe information loss in imaging as shown in Fig. 2a. Coherent speckle induces a large phase fluctuation in the spatial domain but this speckle effect can be far suppressed by tailoring the illumination wavefronts to the target specimen; a representative method for the speckle reduction is shown in Fig. 2b. By controlling the incoming wavefronts over time, the coherent speckles in the spatial domain can be averaged out during the CCD exposure time in the time domain. Multiple coherent beams with different propagation vectors are focused to the sample so the scattered and diffracted wavefronts can be washed out. In order to realize this concept with parametric tunability, we introduced an electroactive optical diffuser having a large number of oscillating scattering particles (at 300 Hz) on the transparent rotating base. An electroactive optical diffuser comprises of a static diffuser (SD) and a rotationally moving diffuser (MD) with a specific diffusion angle (DA). By controlling the particle size, surface density, and rotation speed, the background speckle level and spatial coherence can be tuned (particle size: 10, 30, and 100 µm; surface density: 128, 42, and 13 particles per mm^2^). Figure 2c shows the conceptual diagram of the spatial coherence control using an electroactive micro-optic diffuser. Because the diffuser rotates and scatters the impinging collimated laser beam (*λ*_1_, *k*_1_), any single point on the sample experiences a time-varying phase modulation with different propagation vectors (λ_1_, *k*_1_ ∼ *k*_*N*_). Therefore, any point on the image plane will experience the random walk of time-varying field amplitudes and phases. As the result, the speckle intensity at a point on the image plane varies with time. Because an image sensor (i.e. CCD) captures an image over an exposure time, if the phase modulation is fast enough (much faster than the sensor’s exposure time), the intensity variation due to the phase modulation can be effectively averaged out on the imaging sensor. For the non-mechanical rotation of the scatterers, the dielectric elastomer actuator (DEA) was used as the rotational mechanism. The four independent electrodes are installed to obtain the rotational motion as shown in Fig. 2c. Under the activation, the electrodes make the elastomer film around them to expand by the electro-static force so push the particles to the other direction. A smooth rotational motion can be attained by making the four electrodes (as shown as E1, E2, E3 and E4 in Fig. 2c) have the same amplitude and frequency but with a relative phase delay of 90° in between.

Three optical diffusers having different particle sizes were selected for this investigation, diffuser-1: DA = 1°, diffuser-2: DA = 6°, and diffuser-3: DA = 12° (Refer to the detailed specification in Table 1). A diffuser can be regarded as a numerous number of small point sources placed in the illumination path which diffracts the impinging beam. Smaller particle size increases the diffraction angle from the micro-structures. In combination with the temporal phase averaging effect, a spatially extended incoherent source realized by a larger diffraction angle makes the spatial coherence decrease, as described by the van Cittert-Zernik theorem^7^. This results in a large number of uncorrelated speckle patterns, which lowers the speckle noise. In other words, smaller structural size makes a larger number of phasors that independently fluctuate with random phases; this eventually average out with each other over the exposure time. Therefore, the number of point sources per unit area (*N*) contributing to the sample illumination is determined by the spot size on the diffuser; when *N*-point sources contribute the illumination, the speckle contrast decreases by a factor of *N* ^*−1/2*^. The degree of reduction improves by installing additional diffusers in the illumination path. Here, double-diffuser configuration was used by installing one SD and one MD.

**Table 1:** Specification of scattering films used for speckle reduction.

	SD/MD, DA = 1°	SD/MD, DA = 6°	SD/MD, DA = 12°
Particle size (μm)	100	30	10
Particle surface density (for single diffuser) (#/mm^2^)	13	42	128
Diffusion angle of each diffuser	0.7° (MD) 0.7° (SD)	4.2° (MD) 4.2° (SD)	8.5° (MD) 8.5° (SD)
Diffusion angle of the overall diffuser (SD/MD)	1°	6°	12°
Effective aperture (mm)	5	5	5

### Speckle Reduction in Non-Coherent Bright-Field Laser Imaging

**Figure 3:**
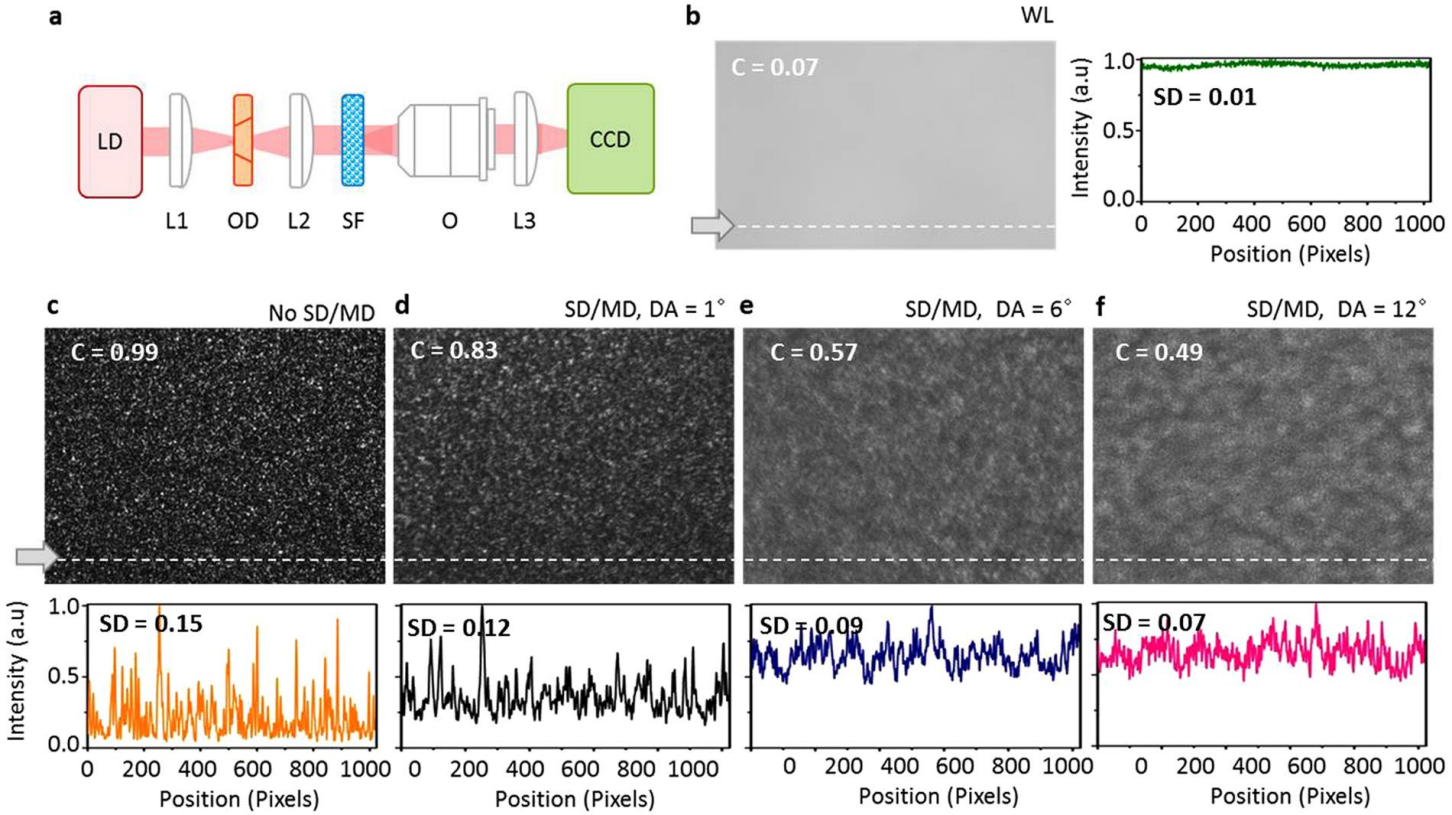
Speckle reduction by introducing optical diffusers in illumination path. (**a**) Optical configuration. Scattering film was imaged with five different illuminations, by (**b**) white light (WL), (**c**) a CW laser, (**d**) a CW laser, with a static diffuser (SD) and moving diffuser (MD) in the illumination path with a diffusion angle (DA) of 1°, (**e**) a CW laser, SD/MD, DA = 6°, and (**f**) a CW laser, SD/MD, DA = 12°. The intensity line profiles and standard deviation (SD) of the images are shown below. LD: laser diode, L: lens, OD: optical diffuser, SF: scattering film, O: objective lens, and CCD: charge coupled device.

To demonstrate the low spatial coherence of a continuous wave (CW) laser under the proposed speckle reduction method, a comparative experiment was carried out with and without the optical diffusers as well as a broadband halogen lamp source, as an ideal source for speckle-free imaging. Imaging tests were conducted in transmission mode under Kohler illumination, as shown in Fig. 3a. As the first step, no imaging object was installed on the object plane for the background check; thus, the light from the source passes through a highly scattering film (DG10-600-MD, Thorlabs) and directly imaged on the CCD. The background speckle images taken with five illumination conditions are shown in Fig. 3b–f. For quantitative comparison, for each case, we calculated the speckle contrast of the image and also the standard deviations (SD) of the intensity line profile. The speckle contrast, *C*, was calculated as $C=S{D}_{I}/I$, where *SD*_*I*_ is the standard deviation of the intensity, and *I* is the average intensity for each image. In the case of broadband Tungsten-Halogen light source, the speckle noise was imperceptible, which was quantified to the speckle contrast of 0.07 and SD of 0.01 (See Fig. 3b). When a high-brightness CW laser was used as the light source, serious coherent speckles were detected as shown in Fig. 3c, providing the speckle contract of 0.99 and SD of 0.15. To avoid the CCD saturation, the overall intensity was intentionally attenuated. For best quality imaging, the coherent background speckle noise in laser imaging should be far suppressed so that clear images can be provided with a high brightness of the laser illumination. Figure 3d–f show the images under the tunable speckle reduction by introducing the optical differs in the illumination path. The speckle contrast decreases as the DA of the diffuser increases, from 0.99 without the diffusers to 0.83, 0.57, and 0.49 with increasing diffusion angle; SD decreases from 0.15 to 0.12, 0.09 and 0.07 in the meantime. By controlling the particle size and DA of the optical diffusers, we were able to tailor the spatial coherence down hence the speckle contrast.

**Figure 4:**
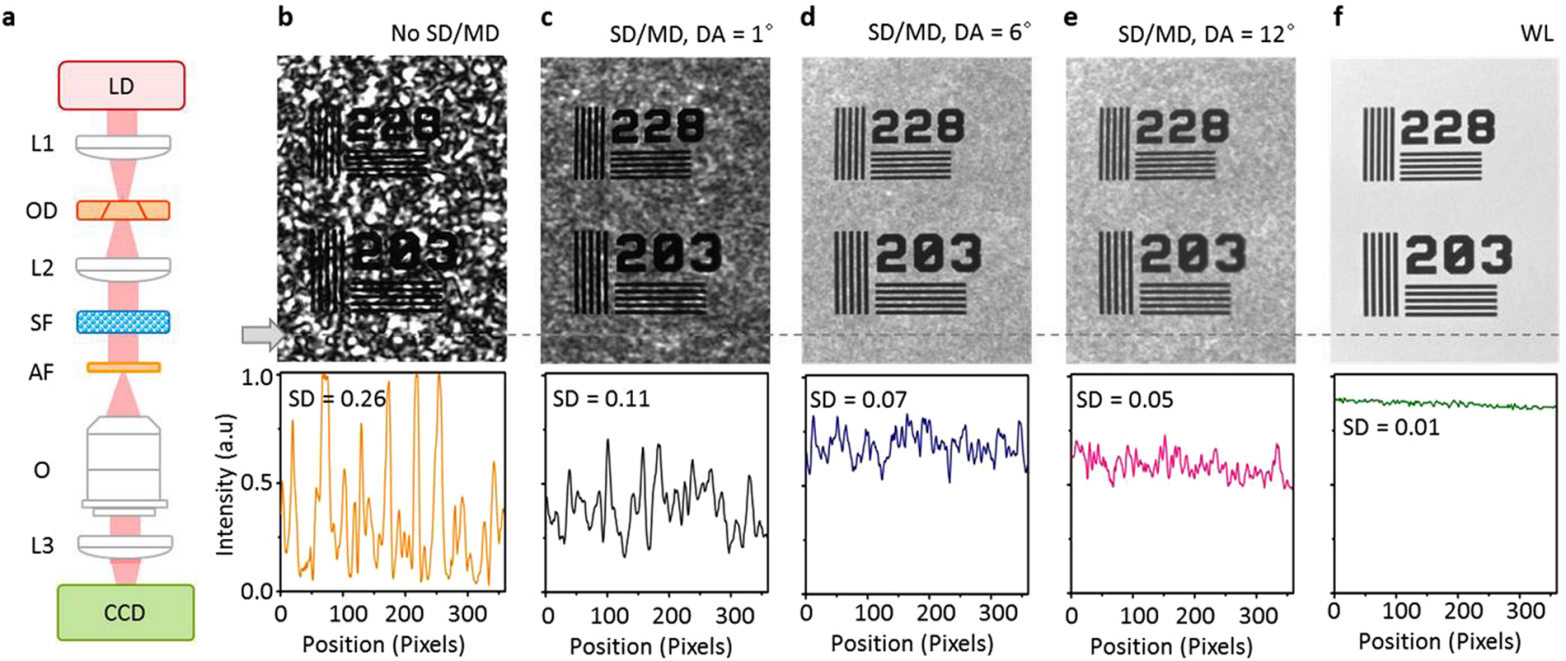
Speckle reduction in optical imaging. (**a**) Optical configuration. 1963A test chart was imaged with a scattering film under five different illuminations, (**b**) a CW laser, (**c**) a CW laser, with a static diffuser (SD) and moving diffuser (MD) in the illumination path with a diffusion angle (DA) of 1°, (**d**) a CW laser, SD/MD, DA = 6°, and (**e**) a CW laser, SD/MD, DA = 12°, and (**f**) white light (WL). The intensity line profiles and standard deviation (SD) of the images are shown below. LD: laser diode, L: lens, OD: optical diffuser, SF: scattering film, AF: air force test target, O: objective lens, and CCD: charge coupled device.

In order to demonstrate the improved image quality with the speckle reduction, a 1963A US Air Force (AF) resolution test chart was imaged under the five illumination conditions as shown in Fig. 4. The scattering film was placed on the illumination side of the AF chart to simulate the random particles in the optical microscope systems, such as biological tissues, plasmonic nanoparticles, and non-ideal optics with surface roughness or contamination. The optical configuration in Fig. 4a is equivalent to imaging system for the rough surfaces^8^. Under the spatially coherent CW laser illumination, the complicated background speckle patterns overlaps with the AF chart image as shown in Fig. 4b; even with the high brightness of CW lasers, this severe image contamination has hampered the wide use of CW laser as the light source for the optical imaging with scatters inside. With the aid of the optical diffusers, the speckle noise was far suppressed so cleaner object images could be recorded as shown in Fig. 4c–e; as DA increases, the quality of the images improves, which was quantified in SD from 0.26 to 0.11, 0.07, and 0.05. Although the image is still cleaner in the case of using the broadband halogen lamp as shown in Fig. 4f, note that a CW laser provides a highly bright illumination and a high temporal coherence which can be used for optical interferometry.

### Speckle Reduction in Laser Interferometry for Quantitative Phase Analysis

**Figure 5:**
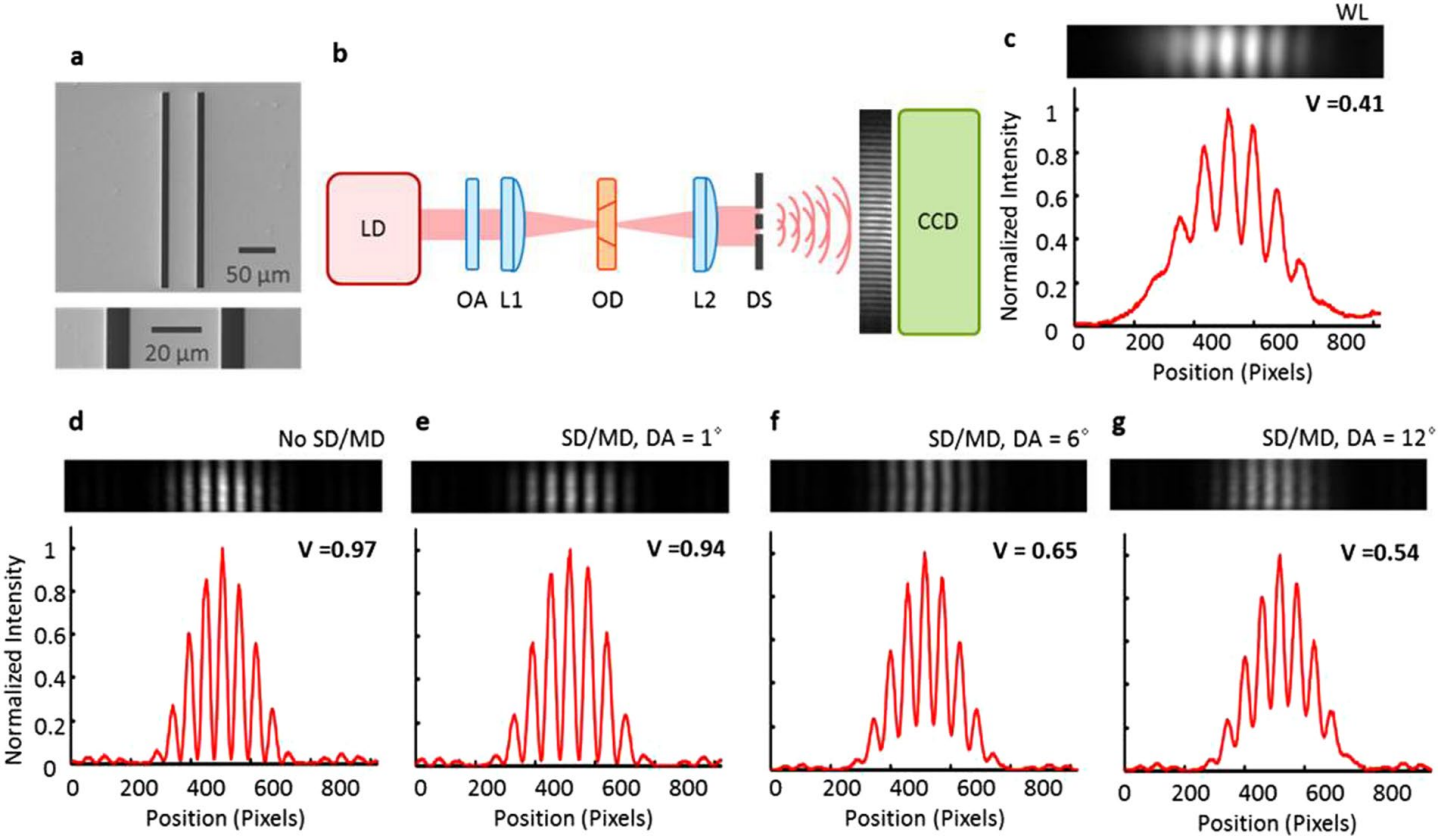
Spatial coherence measurement by Young’s double slit experiment. (**a**) SEM images of the double slit. (**b**) Optical configuration. Young’s double slit interferograms were recorded under five different illuminations, (**c**) a white light (WL), (**d**) a CW laser, (**e**) a CW laser, with a static diffuser (SD) and moving diffuser (MD) in the illumination path with a diffusion angle (DA) of 1°, (**f**) a CW laser, SD/MD, DA = 6°, and (**g**) a CW laser, SD/MD, DA = 12°, The normalized intensity line profiles are shown below. LD: laser diode, OA: optical attenuator, L: lens, OD: optical diffuser, DS: double slit, and CCD: charge coupled device.

In this investigation, we are trying to reduce the coherent speckles with the aid of optical diffusers. However, coherence is highly important in quantitative studies of cellular and material properties using optical interferometry. For example, the detailed cellular properties such as height, volume, homogeneity, cytoplasm’s distribution, and vibrational modes can be determined using QPI using a CW laser as the light source. Therefore, we tried to check whether the coherence can be still preserved even under the proposed speckle reduction. Young’s double slit experiment was introduced here, which is a well-known method for characterizing the spatial coherence of the light sources. For the purpose, a double slit was fabricated with 10 μm width and 50 μm separation (See Fig. 5a) by the focused-ion-beam process at a 150-nm thick gold thin-film on top of a single crystal sapphire substrate. A 15-nm thin titanium layer was located between the gold and sapphire as the adhesive layer; chemical vapor deposition was used to coat the gold by titanium layers. Light from a CW laser transmitted through the optical diffuser and then collimated by a plano-convex lens (See Fig. 5b). The collimated beam was incident on the double slit and the resulting two diffracted beams propagated though the free space, which is long enough for the far-field Fraunhoffer diffraction. The two diffracted beams made interference at a CCD as shown in Fig. 5b. The visibility of the interference pattern at the CCD provides a measure of the spatial coherence. For comparison, we first measured the spatial coherence of the broadband light source (Tungsten-Halogen light source) as the reference as shown in Fig. 5c. Then, the spatial coherence of a CW laser under the speckle reduction by different optical diffusers were evaluated as shown in Figs. 5d–g. The visibility of the interferogram shows the spatial coherence of the light source, whereas the broad envelope in the shape of sinc^2^ function is dominated by the diffraction due to the finite slit width. For quantitative description of the spatial coherence, we obtained the mutual coherence function (γ) from the interference fringes. The degree of coherence between two electric field, E_1_ and E_2_, is defined as ${E}_{1}{E}_{2}^{\ast }/\sqrt{{I}_{1}{I}_{2}}$, where ${I}_{1}={|{E}_{1}|}^{2}$, and ${I}_{2}={|{E}_{2}|}^{2}$. In our coherence measurement, the intensity on the two slits is equal and γ reduces to the visibility γ = *V* = (*I*_*max*_ − *I*_*min*_)/(*I*_*max*_ + *I*_*min*_), where *I*_*max*_ and *I*_*min*_ are the maximum and minimum intensities of the interference fringes. The reference visibility with the broadband light source was 0.41. Compared to this, the visibility with a coherent CW laser under different diffusers decreased from 0.97 to 0.94, 0.65, and 0.54 with the increased DA from 0° to 1°, 6°, and 12°. This implies that one can actively tune the spatial coherence over a wide tunable range so that the visibility can be controlled from 0.54 to 0.94 with the aid of the optical diffusers; 0.97 is the maximum visibility that can be realized with a highly coherent CW laser and 0.41 is the minimum visibility attained by an incoherent broadband light source. Therefore, the spatial coherence of a coherent laser can be optimized to satisfy the requirements by different microscope systems, so that the background speckle noise can be efficiently suppressed while preserving the visibility of the interferograms for the quantitative phase analysis.

**Figure 6:**
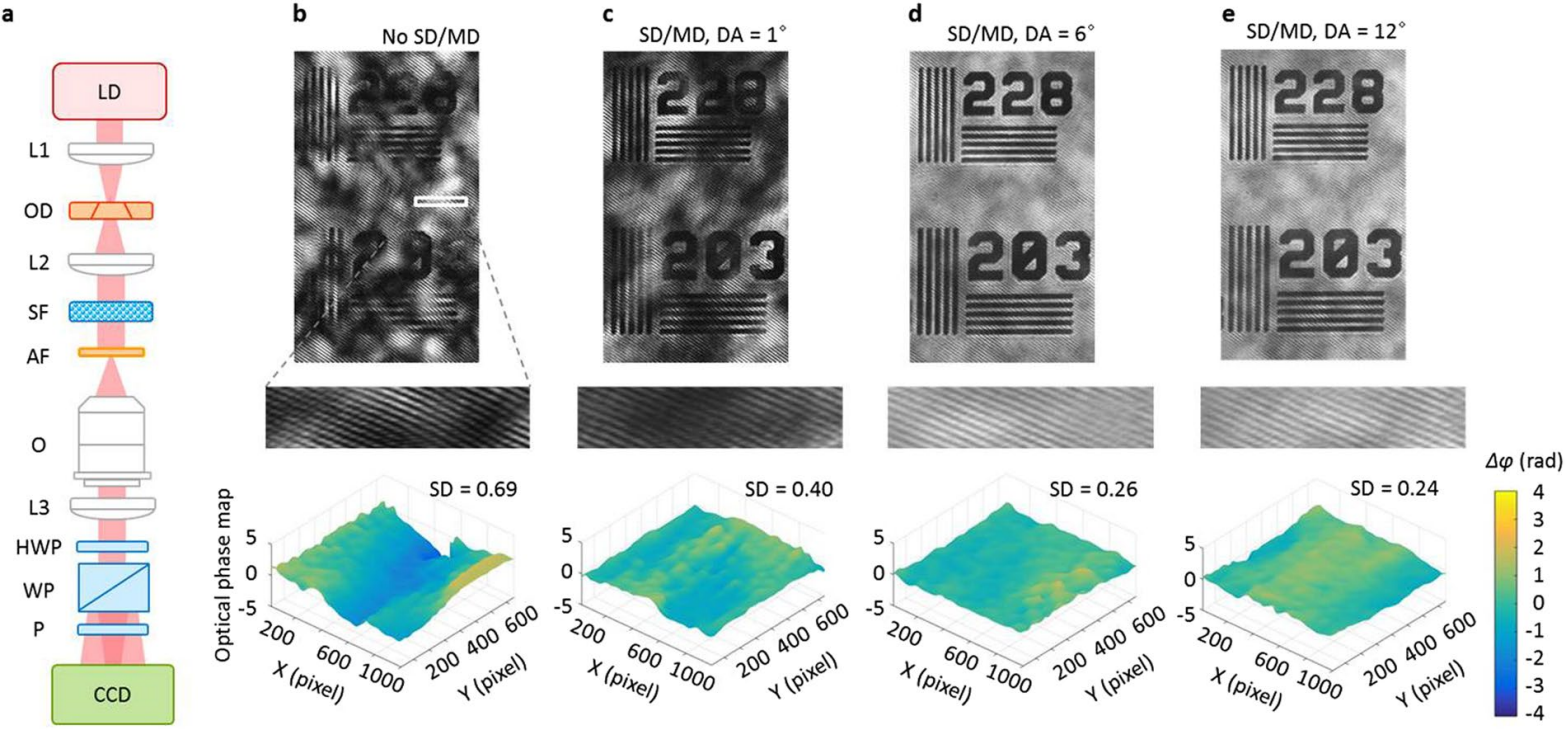
Coherent interferometric phase imaging with speckle reduction. (**a**) Optical system for off-axis common-path QPI with speckle reduction. 1963A US AF test chart was imaged after a biologically relevant scattering film under four different illuminations, (**b**) a CW laser, (**c**) a CW laser, with a static diffuser (SD) and moving diffuser (MD) in the illumination path with a diffusion angle (DA) of 1°, (**d**) a CW laser, SD/MD, DA = 6°, and (**e**) a CW laser, SD/MD, DA = 12°. Reconstructed optical phase maps of the cropped areas are shown below. LD: laser diode, L: lens, OD: optical diffuser, SF: scattering film, AF: air force test chart, O: objective lens, HWP: half wave plate, WP: Wollaston prism, P: polarizer, and CCD: charge coupled device.

Coherent interferometric imaging over a FOV with successful speckle reduction was demonstrated in Fig. 6 by imaging a 1963A US AF test chart using off-axis common-path QPI^25^. A biologically relevant scattering film with a small diffusion angle of 1° in full-width-half-maximum (holographic diffuser from Edmund Optics, #47–991) was placed in the illumination side of the test chart to provide random phase delays to the impinging light as shown in Fig. 6a, which resulted in speckle illumination to the object. An objective lens (NA = 0.45, M = 20) was installed for the imaging in the inverted microscope. QPI part is composed of three optical components: a half-wave plate, a Wollaston prism, and a linear polarizer. The vertically polarized beam passes through the half-wave plate, resulting in the polarization rotation by 45°. Then, the Wollaston prism splits the beam into two beams with different propagation directions by the polarization state. The linear polarizer located after the Wollaston prism is aligned to 45° with respect to two split beams, which makes the two beams to interfere with each other at the image plane, where a CCD camera records the interferogram. The half-wave plate can be used for balancing the intensities at the two polarization states, which results in high visibility of the interferogram.

We successfully obtained QPI interferograms of the AF test chart under different coherent CW laser illuminations with and without the optical diffusers as shown in Figs. 6b–e. Note that these interferograms cannot be attained with a white light illumination because the polarization-dependant phase delay in Wollaston prism is longer than the short coherence length (a few micrometres) of the broadband white light sources. As the DA increases, the interferogram gets cleaner with suppressed speckle noise; this trend is more evident at zoom-in images. To quantify the speckle reduction, the quantitative phase analysis was performed by the fast Fourier transform (FFT) analysis^26,27^, and a topographic phase map of the cropped area (shown as white rectangle in Fig. 6b) was reconstructed. The standard deviation of the spatial phase noise decreases from 0.69 rad (without the optical diffuser) to 0.24 rad (with SD/MD, DA = 12°), as shown in Fig. 6b–e. Furthermore, the global low-spatial-frequency background phase distortion shown in Fig. 6b was removed in Fig. 6e; because this US AF test chart is a well-defined sample with a uniform thickness, there should not be global phase distortion. Therefore, the speckle reduction improves the measurement precision and sensitivity in QPI^28,29^. These results show that our approach suppressed the speckle noise well so that the standard deviation of the phase noise decreased by 2.9 times with a background phase distortion removal. The speckle reduction in QPI for real phase objects (polystyrene beads and breast cancer cells) was recently demonstrated to suppress the coherent artifacts and enhance the lateral resolution^6^. This proposes the application of this speckle reduction technique in revealing small biological, plasmonic, and optical objects in complex scattering medium over a larger FOV by an interferometric QPI.

## Conclusion

We suppressed the background speckle noise in high-brightness coherent interferometric and non-interferometric optical imaging by manipulating the spatial coherence of the lasers using tunable electroactive micro-optic diffusers. The optimal control of the spatial coherence was realized by tuning the particle size, surface density, and rotation speed of the electroactive optical diffusers. The spatial coherence of the laser was tailored down from 0.94 (for no SD/MD) to 0.54 (for SD/MD, DA = 12°) in visibility, which resulted in suppression of the speckle noise by factor of two. This method enables an efficient way of acquiring high-brightness, speckle-free, clean images without subsequent image processing. Dynamic phase noise induced by the background speckle was reduced from 0.69 rad (for no SD/MD) to 0.24 rad (for SD/MD, DA = 12°) by 2.9 times. This dynamic phase noise suppression over a large field-of-view improves the measurement precision of quantitative phase imaging, so enables efficient reconstruction of 3D height and refractive index profiles of biological, medical, and industrial samples. Our spatial coherence control and speckle reduction techniques will lead to a wide range of incoherent and coherent laser imaging requiring a fast, bright, clean, and phase-stable data acquisition over a large field-of-view.
